# Simulation and Experimental Study of Laser Processing NdFeB Microarray Structure

**DOI:** 10.3390/mi14040808

**Published:** 2023-03-31

**Authors:** Yong Zhao, Shuo Wang, Wenhui Yu, Pengyu Long, Jinlong Zhang, Wentao Tian, Fei Gao, Zhuji Jin, Hongyu Zheng, Chunjin Wang, Jiang Guo

**Affiliations:** 1 State Key Laboratory of High-Performance Precision Manufacturing, Dalian University of Technology, Dalian 116024, China; 2 Centre for Advanced Laser Manufacturing (CALM), School of Mechanical Engineering, Shandong University of Technology, Zibo 255000, China; 3 State Key Laboratory of Ultra-Precision Machining Technology, Department of Industrial and Systems Engineering, The Hong Kong Polytechnic University, Hong Kong, China

**Keywords:** NdFeB, laser processing, simulation, microstructure formation mechanism, melt pool flow evolution

## Abstract

NdFeB materials are widely used in the manufacturing of micro-linear motor sliders due to their excellent permanent magnetic properties. However, there are many challenges in processing the slider with micro-structures on the surface, such as complicated steps and low efficiency. Laser processing is expected to solve these problems, but few studies have been reported. Therefore, simulation and experiment studies in this area are of great significance. In this study, a two-dimensional simulation model of laser-processed NdFeB material was established. Based on the overall effects of surface tension, recoil pressure, and gravity, the temperature field distribution and morphological characteristics with laser processing were analyzed. The flow evolution in the melt pool was discussed, and the mechanism of microstructure formation was revealed. In addition, the effect of laser scanning speed and average power on machining morphology was investigated. The results show that at an average power of 8 W and a scanning speed of 100 mm/s, the simulated ablation depth is 43 μm, which is consistent with the experimental results. During the machining process, the molten material accumulated on the inner wall and the outlet of the crater after sputtering and refluxing, forming a V-shaped pit. The ablation depth decreases with the increment of the scanning speed, while the depth and length of the melt pool, along with the height of the recast layer, increase with the average power.

## 1. Introduction

Micro-linear motors are widely used in microrobots, smartphones, and medical applications. The relative motion between the slider with the microarray structure and the stator with the embedded micro-coil is generated by the Lorentz force, which drives the micro-linear motor to produce linear micro-movements. NdFeB materials are used in the manufacturing of sliders for micro-linear motors because of their excellent permanent magnetic properties. Currently, micro-cutting and micro-milling technologies are used to manufacture micro-array structures on the surface of NdFeB, which are then used to produce the sliders required for micro-linear motors. In addition, micro-electro-mechanical systems (MEMS) technology is also used in the manufacture of sliders. Zhi et al. [1] bonded NdFeB sheets to a silicon substrate and cut it into a long strip pattern at 100 µm intervals by using a cutting saw. Furthermore, Wang et al. [2] fabricated a chessboard microarray structure with a pitch of 1.2 mm and a length and width of 810 µm on the surface of a NdFeB sheet by lithography and wet etching. These reported processing methods can process regular arrays with hundreds of microns scale and misaligned arrays with the millimeter scale. With the miniaturization of micro-linear motors, it is desired to develop the efficient processing technologies that can manufacture smaller-scale arrays on NdFeB sheets. The micro-cutting and micro-milling technologies can process regular arrays of 100 µm, but it is difficult to fabricate misaligned arrays of the same scale. Moreover, the processing efficiency is low. Although MEMS can fabricate misaligned array structures, it is cumbersome and inefficient, so it is not suitable for processing deeper structures.

Laser processing technology [3,4] is widely used in preparing the functional surface and treating artificial bone due to its controllability and high efficiency. Li et al. [5] used picosecond pulsed lasers to generate precise mesh structures on pure titanium surfaces to improve their surface wettability and biocompatibility. Melo-Fonseca et al. [6] fabricated pyramidal-shaped microscale columns on metallic biomaterials, thereby improving the interaction between bone and implant. Zhao et al. [7] investigated the feasibility of preparing a bionic superhydrophobic stainless steel surface by laser precision engineering to achieve dynamic control of targeted superhydrophobicity and water transport. The above applications demonstrate the potential of laser processing technology to process microscale array structures on NdFeB surfaces. Currently, there are few studies on the laser processing of NdFeB materials. Numerical simulations can greatly reduce the number of experiments and facilitate the investigation of the material removal mechanism during laser processing. In terms of simulation, Lim et al. [8] investigated the effect of processing parameters on the ablation depth and morphology during nanosecond pulsed laser processing of copper and aluminum by finite element simulations. Berenyi et al. [9] investigated the heat effect induced during the laser processing of copper materials by building a three-dimensional finite element model. Zhang et al. [10] carried out finite element modeling of the nanosecond pulsed laser processing of stainless steel, and the moving boundary conditions were considered. Considering the solid–liquid phase transition and the removal of the materials due to liquid evaporation is significant in simulating the material removal process. The surface tension caused by the flow field and the Marangoni effect caused by the tension gradient also have a large impact on the simulation of the laser processing process. Ding et al. [11] considered the Marangoni effect as the main driving force for fluid flow from the top center region of the melt pool to the outside of the rim in selective laser melting (SLM) modeling. Similarly, Yuan et al. [12] suggested that in SLM, the Marangoni effect plays a key role in enhancing convective heat transfer and changing the melt pool geometry. In addition to the Marangoni effect, the center of the molten pool is mainly subject to recoil pressure, which drives surface profile changes. Cho et al. [13] investigated the molten pool dynamics of laser welding and concluded that molten metal flow is mainly driven by surface tension and that the buoyancy drive in the bulk forces cannot be neglected. The energy of the nanosecond pulsed laser is high; the recoil pressure is the main driving force for the formation of the crater profile. Yuan et al. [14] carried out simulations and experiments on the melt pool state during SLM and found that the effect of recoil pressure was more significant when the scanning speed became lower. Material loss due to evaporation during laser processing should not be ignored either. Zhang et al. [15] considered the recoil pressure and mass loss due to material evaporation in the model and investigated the effect of laser energy and pulse width on the morphology and quality of the processed micro-hole. Therefore, it is necessary to consider the effect of fluid flow and recoil pressure when simulating the effect of laser processing parameters on the processed morphology. There are two main methods for describing the melt pool surface: the surface tracing method, represented by the level set method, and the free deformation method, represented by the deformation geometry method. Zhu et al. [16] developed both deformation geometry and level set models and demonstrated through comparison that both methods were suitable for laser processing melt pool simulation, while the deformation geometry method was more accurate and efficient. The level set method has a unique advantage in modeling the two-phase flow direction, while the deformation geometry method is preferred as the gas–liquid phase change is not considered.

In this study, the COMSOL finite element analysis software was used to couple the physical fields of heat transfer and fluid flow, and a 2D simulation model of laser processing of NdFeB materials was established. The model considers heat conduction, heat convection, and evaporation heat loss, as well as the effects of surface tension, recoil pressure, and gravity on the fluid. The ablation rate was added for evaporation removal by introducing the Hertz–Knudsen equation via the deformation geometry method. The temperature field distribution, morphological characteristics, and the flow field velocity of the melt pool were analyzed. In addition, the effects of laser scanning speed and average power on processing quality were simulated. The accuracy of the model was verified by conducting experiments with a nanosecond laser using the same parameters as in the simulation for microarray processing. Based on this research, the heavy laser processing experiment will be replaced by simulations, and then the processing parameters can be inverted according to the simulation results. This can greatly reduce the number of experiments and help to obtain the preferred processing parameters.

## 2. Numerical Simulation Modeling

The COMSOL Multiphysics simulation software has complete finite element simulation capabilities and has significant advantages in multiphysics field coupling. It was chosen to simulate the process of laser processing of NdFeB and the material removal mechanism. The physical fields of heat transfer, fluid flow, and deformation geometry were coupled in this study. The physical parameters of the required NdFeB material are shown in Table 1, and the following assumptions were made to simplify the model.

1. The laser has a Gaussian profile. The ablation process is stable, and the reflection of the laser beam on the surface of the crater is neglected.
2. Solid metals are considered very viscous fluids, and molten metals are incompressible non-Newtonian fluids under laminar flow.
3. Effects such as the shielding effect of the plasma are ignored.

### 2.1. Heat Transfer Modeling

According to the assumptions, the laser source is a Gaussian surface heat source. The pulse function p(t) was set, which in turn set the pulse width, frequency, and period of the laser. With respect to the direction x, the laser energy density function Q(x,t) at time t can be expressed as Equation (1):

(1) $$Q\left(x,t\right)=(\frac{2P}{\pi r^{2}})exp(-\frac{2{\left(x-v_{0}t\right)}^{2}}{r^{2}})$$

where P is the power of the laser, r is the laser spot diameter, and $v_{0}$ is the spot travel speed.

At this point, the heat flux $q_{0}$ at the input boundary can be expressed as Equation (2):

(2) $$q_{0}=a\cdot Q(x,t)\cdot p(t)$$

where a is the absorption rate of the material at the laser wavelength. To find the exact absorption rate parameter, the law of variation with temperature was considered, as shown in Equation (3) [17]:

(3) $$a(T)=\sqrt{\frac{4\pi c\varepsilon_{0}\left[1+\alpha (T-T_{a})\right]}{\lambda \sigma_{0}}}$$

where $\varepsilon_{0}$ is the permittivity of vacuum, α is the resistance coefficient of the target as a function of temperature, λ is the wavelength of the laser beam, c is the velocity of light, and $\sigma_{0}$ is the target conductance at the initial temperature $T_{a}$.

When the laser acted on the surface of the material, the material absorbed energy and heated up rapidly, and a phase transition occurred, which follows the equation of energy conservation, as shown in Equation (4):

(4) $$\rho C_{p}(\frac{\partial T}{\partial t}+u\cdot \nabla T)-\nabla \cdot (k\nabla T)=q_{0}$$

where ρ is the liquid phase density, $C_{p}$ is the specific heat, u is the fluid flow rate, and k is the thermal conductivity.

The materials undergo phase transitions, including melting, evaporation, and sublimation, during laser processing. This process will absorb or release a lot of latent heat so that the temperature at the phase transition point is constant, and the latent heat of phase transition needs to be considered. The equivalent heat capacity method allows the treatment of the latent heat energy as an equivalent heat capacity, where the heat capacity changes considerably in the interval near the phase transition point, and the area enclosed by the heat capacity vs. temperature graph is the latent heat of phase transition [18]. As shown in Equation (5),

(5) $$C_{p}=C_{p0}+\delta_{m}L_{m}+\frac{L_{m}}{T_{m}}H^{\prime }\;((T-T_{m}),\Delta T)+\delta_{v}L_{v}+\frac{L_{v}}{T_{v}\;}H^{\prime }\;((T-T_{v}\;),\Delta T)$$

where $\delta_{m}$ and $\delta_{v}$ are temperature smoothing functions for the phase transition interval, with expressions as shown in Equations (6) and (7), respectively:

(6) $$\delta_{m}=\frac{\exp[-\frac{{\left(T-T_{m}\right)}^{2}}{\Delta T^{2}}]}{\Delta T\sqrt{\pi }}$$

(7) $$\delta_{v}=\frac{\exp[-\frac{{\left(T-T_{v}\right)}^{2}}{\Delta T^{2}}]}{\Delta T\sqrt{\pi }}$$

$L_{m}$ and $L_{v}$ denote the latent heat of melting and the latent heat of vaporization, respectively, $T_{m}$ and $T_{v}$ denote the melting temperature and vaporization temperature, $H^{\prime }\;$is the Heasivide function, and ΔT is the phase transition half interval temperature.

### 2.2. Heat Transfer Boundary Condition

The nanosecond pulsed laser processing also involves convective heat transfer, radiative heat transfer, and evaporative heat loss. The above heat losses were considered at the processing surface boundary, as shown in Equation (8):

(8) $$-k\nabla T=h_{1}(T-T_{a})+\varepsilon \sigma (T^{4}-T_{a}^{4})+q_{v}$$

where $h_{1}$ is the heat transfer coefficient, ε is the emissivity, σ is the Stefan–Boltzmann constant, and $q_{v}$ is the evaporative heat loss; the specific expression is shown in Equation (9) [19].

(9) $$q_{v}=\dot{m}L_{v}$$

(10) $$\dot{m}=(1-\beta )\sqrt{\frac{M_{mol}}{2\pi R_{0}T}}P_{0}\exp(\frac{L_{v}M_{mol}}{R_{0}}(\frac{1}{T_{v}}-\frac{1}{T}))$$

(11) $$P_{a}(T)=P_{0}\cdot \exp(\frac{M_{mol}L_{v}}{R_{0}}(\frac{1}{T_{v}}-\frac{1}{T}))$$

where $\dot{m}$ is the evaporation rate, $M_{mol}$ is the molar mass of the metal, $P_{a}(T)$ represents the saturation vapor pressure, β is the diffusion coefficient, $R_{0}$ is the universal gas constant, and $P_{0}$ is the atmospheric pressure.

### 2.3. Solid–Liquid Interface Treatment

The liquid phase fraction θ was required to treat each parameter as a temperature-dependent segmental function bounded by the solid–liquid phase temperature line, as shown in Equation (12).

(12) $$\theta =\left\{ \begin{array}{ll} 0 & \;,T<T_{m}-\Delta T\\ \frac{T-(T_{m}-\Delta T)}{2\Delta T} & ,\;T_{m}-\Delta T<T<T_{m}+\Delta T\\ 1\; & ,\;T>T_{m}-\Delta T \end{array}\right.$$

At the same time, the phase transition will lead to a change in the physical parameters of the material. Therefore, the transition parameters need to be smoothed in the transition zone of the solid–liquid interface. The density, thermal conductivity, and kinetic viscosity of the transition zone are expressed in Equations (13)–(15), respectively.

(13) $$\rho =(1-\theta )\rho_{s}+\theta \rho_{l}$$

(14) $$k=(1-\theta )k_{s}+\theta k_{l}$$

(15) $$\mu =(1-\theta )\mu_{s}+\theta \mu_{l}$$

where $\rho_{s}$ and $\rho_{l}$ denote the density of solids and liquids, respectively, $k_{s}$ and $k_{l}$ denote the thermal conductivity of solid phase and liquid phase, respectively, $\mu_{s}$ and $\mu_{l}$ are dynamic viscosity of solid phase and liquid phase, respectively.

According to the Carman–Koseny formula [20], a force term was introduced to absorb the loss of momentum in the solid material, as shown in Equation (16).

(16) $$F_{D}=\frac{A_{m}{\left(1-\theta \right)}^{2}}{\theta^{3}+\xi }u$$

where $A_{m}$ is the Carman–Koseny coefficient. The solid material has a large viscous drag, and a larger value needs to be set. In addition, to avoid a zero denominator, a very small amount ξ should be added.

### 2.4. Flow Modelling

The NdFeB material is melted to form a molten pool, and the flow of molten metal in the pool is controlled by the conservation of momentum equation, with the expression shown in Equation (17) [21].

(17) $$\rho \frac{\partial u}{\partial t}+\rho u\cdot \nabla u=\nabla (\mu (\nabla u+{\left(\nabla u\right)}^{2}-pI))+\rho g-\beta_{1}(T-T_{a})\rho g-F_{D}$$

where ρg and $\beta_{1}(T-T_{a})\rho g$ represent the forces of gravity and buoyancy, respectively.

The molten material flowing in the melt pool is subjected to recoil pressure and surface tension, which drive the formation of craters. The recoil pressure was generated by the material evaporation in the opposite direction of evaporation, resulting in driving the liquid materials outwards, as shown in Equation (18) [22]:

(18) $$P_{r}=\left\{ \begin{array}{ll} P_{0} & ,T_{a}\leq T<T_{v}\\ \frac{1+\beta }{2}P_{a}(T) & ,T\geq T_{v} \end{array}\right.$$

Surface tension acts on the surface of molten materials to cause liquid flow, which is also known as Marangoni convection. It is caused by a surface tension gradient due to a free surface temperature gradient, which drives the penetration and expansion of the melt pool. In the central region of the Gaussian surface heat source, where the temperature is highest and the surface tension is lowest, the fluid flows from the center to the edge of the melt pool. The surface tension depended on the material and the temperature, as shown in Equation (19) [23]:

(19) $$\gamma =\gamma_{m}-A_{\gamma }(T-T_{m})-R_{g}T\Gamma_{s}\ln(1+k_{i}a_{i}e^{\frac{\Delta H_{0}}{R_{g}T}})$$

where γ is the coefficient of surface tension between the molten metal and air, and $A_{\gamma }$ represents the temperature coefficient of surface tension. The third term in Equation (19) is insignificant and can be neglected to simplify the model.

### 2.5. Ablation Deformation Modelling and Meshing

The ablation removal process was simulated by the deformation geometry method. The movement of the upper boundary finite element mesh was controlled by the ablation velocity based on the fluid flow rate and the Hertz–Knudsen equation, which was calculated by the hyperplastic smoothing method. The movement velocity of the mesh is shown in Equation (20).

(20) $$s=u+v-\frac{\dot{m}}{\rho }$$

where u and v represent the lateral and longitudinal flow velocities, respectively.

**Figure 1:**
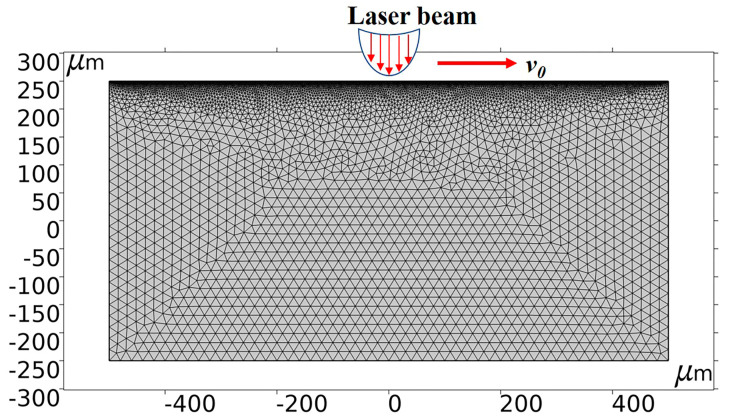
Mesh division image.

The 2D finite element geometry model was generated and shown in Figure 1. The vertical red arrows represent the laser beam loading direction and the horizontal red arrow indicates the laser spot travel direction. The laser beam was initially at the home position and oriented along the positive direction of the *X*-axis. Partitioning was carried out via a free triangular mesh. To improve the model accuracy, the mesh density of the upper boundary was increased. Furthermore, an automatic re-meshing function was used to avoid mesh inversion distortion during the simulation calculations.

## 3. Simulation and Experiment Procedures

For the analysis of the processing morphology, temperature field distribution, and flow fields velocity in the simulation, the average power of the nanosecond pulsed laser was set at 8 W, the pulse width at 40 ns, the frequency at 150 kHz, and the scanning speed at 100 mm/s. Due to the complexity of the model calculation, the variation over 15 pulse cycles was simulated. In this study, the laser processing parameters were determined by the simulation results and the laser parameters adjustment range of the laser processing machine. To investigate the effect of laser scanning speed and average power on the processing results, the laser processing parameters selected for the simulation are shown in Table 2; other parameters were kept constant.

**Figure 2:**
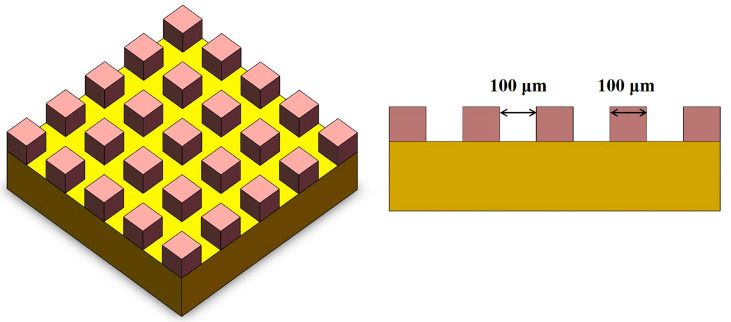
Schematic and dimension of the microarray structure.

The schematic and dimension of the NdFeB microarray structure are shown in Figure 2. The experiments were carried out to verify the accuracy of the model by a UV nanosecond laser. The laser processing parameters used for experiments were consistent with those used in the simulation. After the experiment, the processed microstructures were observed with an ultra-deep field microscope (VHX-600E, Osaka, Japan). In addition, a ZYGO 3D profiler (Zygo 9000, Middlefield, CT, USA) was employed to measure the microstructure and the ablation depth.

## 4. Results and Discussion

### 4.1. Temperature Field and Flow Fields Analysis

**Figure 3:**
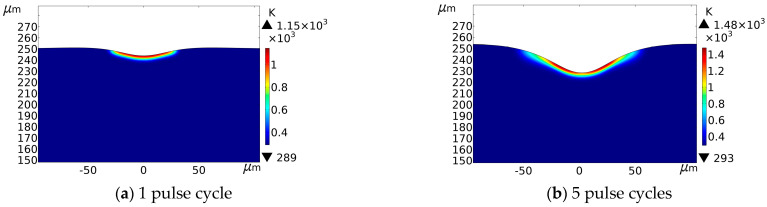

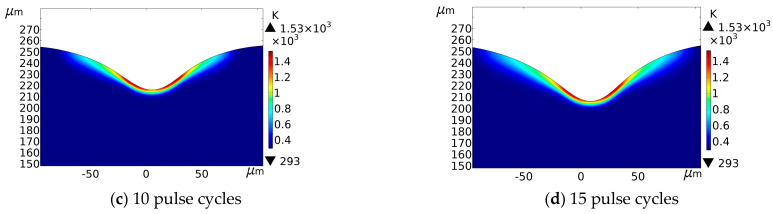
Microstructure morphology and temperature field distribution at different pulse cycles (Average power = 8 W, pulse width = 40 ns, frequency = 150 kHz, scanning speed = 100 mm/s).

Figure 3 shows the formation of the crater and the distribution of the temperature field during the simulation. The NdFeB starts to undergo phase change phenomena such as liquefaction and vaporization under the action of the laser pulse heat source; the material is removed by ablation, the depth and width increase with the increment of laser action time, and a crater is formed under the action of recoil pressure, surface tension, etc., finally causing the molten material to build up at the edges. The figure shows that the temperature at the bottom of the crater was the highest and spread around and inwards to form a heat-affected region. The morphology was roughly symmetrical due to the short travel distance of the laser spot within 15 pulses at a travel speed of 100 mm/s. Due to the nature of the Gaussian distribution of the laser heat source, the crater continues to get deeper as the number of cycles increases, as shown in Figure 3a–d, generating a roughly V-shaped morphology. The phenomenon of material accumulation can be reflected by the dimension of the upper boundary. Comparing the boundaries set in Figure 1, the original height before the simulation was 250 μm. After the laser ablation simulation, the molten material gradually stacked on both sides of the crater, which increased the height to approximately 255 μm, as shown in Figure 3d. The stacking height of the material is about 5 μm.

**Figure 4:**
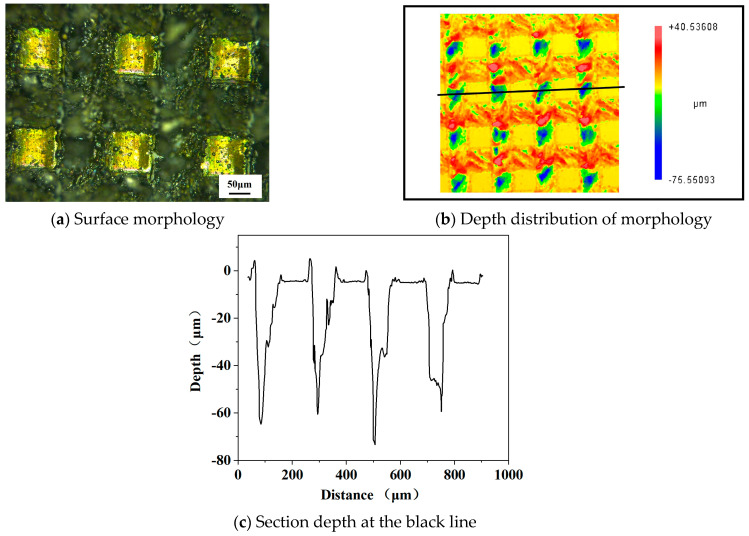
Experimental results of laser processing (Average power = 8 W, pulse width = 40 ns, frequency = 150 kHz, scanning speed = 100 mm/s).

Figure 4a shows the experimental surface morphology of NdFeB with laser processing; the material was stacked at the edge of the columnar array structure. Figure 4c shows the change in section depth at the black line in Figure 4b. As can be seen from the figure, the processed groove has a large taper, and the average depth at the bottom is about 50 μm, while the depth of the simulation result is about 43 μm. In the simulation, due to the limited number of pulses, the scanning distance of the laser beam is short, at a scanning speed of 100 mm/s, and the local heating is insufficient, while the actual processing is multi-pulse and long distance. In addition, the processing experiment is also influenced by multiple factors, so there exists a few micrometers error in depth. Overall, the experiment and simulation results have better consistency in terms of morphology and depth.

**Figure 5:**
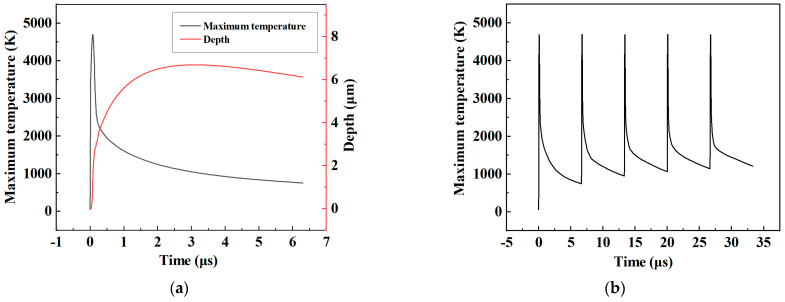
Maximum temperature and ablation depth variation. (**a**) Maximum temperature and ablation depth variation in single pulse cycle. (**b**) Maximum temperature variation in five pulse cycles.

In order to describe the temperature change, the maximum temperature curve was calculated by using a probe to trace the entire process. Figure 5a shows the maximum temperature change and the ablation depth for a single pulse. As can be seen from the figure, the maximum temperature rose rapidly during the pulse duration, and the maximum temperature reached over 4000 K. In this process, the material melting and vaporization occurred. After the process was over, the temperature dropped rapidly and then tended to flatten until the end of the cycle. The change in the ablation depth first increased rapidly, then tended to flatten, and finally showed a slight decrease. The reason of the above phenomenon is that the melting, evaporation, and sublimation of the material occur when the temperature rises sharply. As a result, the material starts to eliminate. When the temperature was below the melting temperature, the molten metal flowed back due to gravity. A recast layer at the bottom of the crater was formed, making the ablation depth drop back. Figure 5b shows the curve of the maximum temperature value within five pulse cycles; it can be seen that the maximum temperature tends to be the same within each cycle, and the minimum temperature gradually rises, which was caused by the heat affected after laser processing.

**Figure 6:**
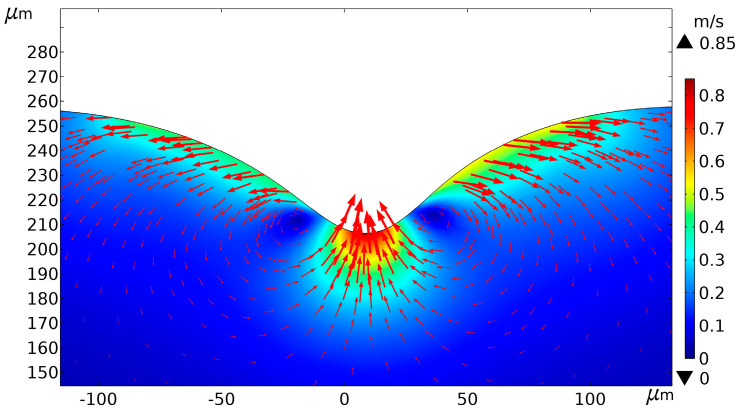
Flow field velocity of the melt pool. (Average power = 8 W, pulse width = 40 ns, frequency = 150 kHz, scanning speed = 100 mm/s).

**Figure 7:**
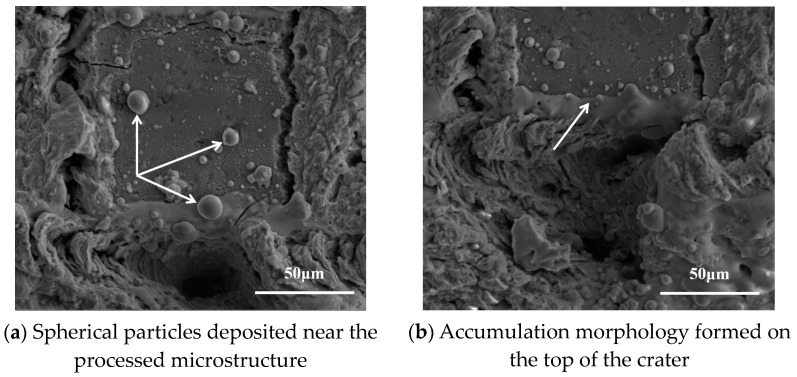
SEM morphology of microstructure after laser processing. (Average power = 8 W, pulse width = 40 ns, frequency = 150 kHz, scanning speed = 100 mm/s).

Figure 6 shows the flow field velocity of the melt pool for 15 pulse cycles. The red arrow in the figure indicates the flow direction, the length of the arrow is proportional to the flow velocity, and the density of the arrow indicates the flow intensity of the flow field. In Figure 6, it can be seen that the direction of flow velocity at the crater bottom was vertically upwards, and the direction of flow velocity at the edge of the crater was approximately the normal direction along the sidewall. The reason is that the crater bottom is the central area of the Gaussian heat source. Under the Marangoni effect, the spontaneous metal liquid flow will occur from the bottom of the high-temperature crater towards the low-temperature sidewalls and the edges of the back. The recoil pressure can then induce a jet of low-viscosity metallic liquid which will split into small droplets during flight to minimize surface tension. This creates the entire spattering process [24]. Figure 7a further demonstrates the ejection phenomenon during processing: the spherical particles deposited near the processed microstructure, as indicated by the white arrows. In contrast, because the temperature on both sides was lower than the vaporization temperature, the molten material flowed roughly along the normal direction of the crater sidewall under the role of the surface tension of the liquid surface and gravity. Eventually, all the molten material solidified, and a recast layer was formed on the inner wall and top of the crater, resulting in morphology as stimulated. As shown by the arrow in Figure 7b, the accumulation morphology formed on the top of the crater when the flowing molten material solidified. The experiment morphology in Figure 7 is consistent with the simulation results in Figure 6. This proves the correctness of the simulation.

### 4.2. Effect of Laser Scanning Speed on the Processing Morphology

The laser scanning speed is an important parameter that affects the processing morphology of the crater. Different laser scanning speeds can produce different spot overlap rates. The spot overlap rate will become small while the scanning speed gets faster. The theoretical overlap rate η between adjacent laser spots can be expressed as Equation (21).

(21) $$\eta =\left(1-\frac{v_{0}}{2fr}\right)\times 100\%$$

where f is the laser pulse frequency, $v_{0}$ is the laser scanning speed, and r is the spot radius.

**Figure 8:**
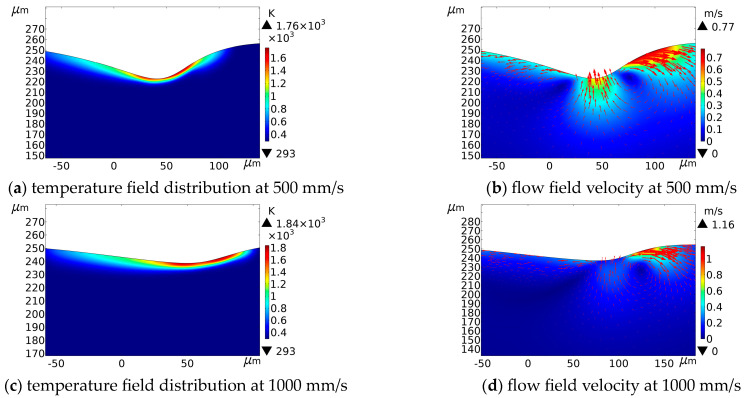
Temperature field distribution and flow field velocity at different scanning speeds (Average power = 8 W, pulse width = 40 ns, frequency = 150 kHz).

The scanning speeds chosen for simulation and experiment were 100 mm/s, 500 mm/s, and 1000 mm/s, respectively, and the spot overlap rates were 96.7%, 83.3%, and 66.7%, respectively. Other laser parameters were kept constant. As the spot scanning speed increased, the linear energy input of the material decreased because the laser-material interaction time decreased. Figure 3d and Figure 6 show the simulation results of temperature field distribution and flow field velocity when the scanning speed is 100 mm/s. Combined with the simulation results in Figure 8, it can be seen that within the 15 pulse cycles, as the scanning speed became faster, the length of the processing area increased, the ablation depth became shallower, and the heat-affected area decreased accordingly. When the scanning speed increased, the maximum flow rate increased, but the flow intensity of the overall area was weakened. The reason of this phenomenon is that, when it was at a larger scanning speed, the residence time of the laser spot at the unit area was short, and the heat-affected zone was small, which made the ablation depth small and flow intensity weak. When the scanning speed was large, the processed area of the crater was farther away from the laser spot, and the flow field velocity became weaker. Moreover, the impact of the recoil pressure on the melt pool was also weakened, so the melt pool flow was relatively gentle, and the ablation depth was reduced, which was conducive to the escape of bubbles from the melt pool. In contrast, the flow field on the right side of the crater was more intense.

**Figure 9:**
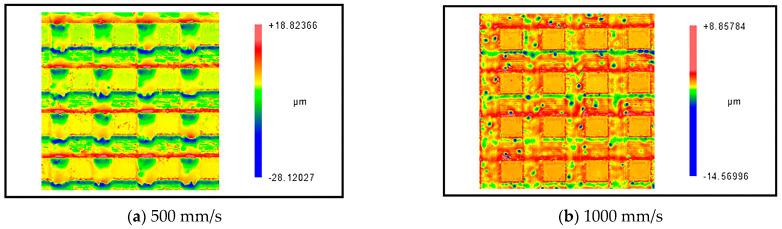
Experimental results of laser processing at different scanning speeds (Average power = 8 W, pulse width = 40 ns, frequency = 150 kHz).

**Figure 10:**
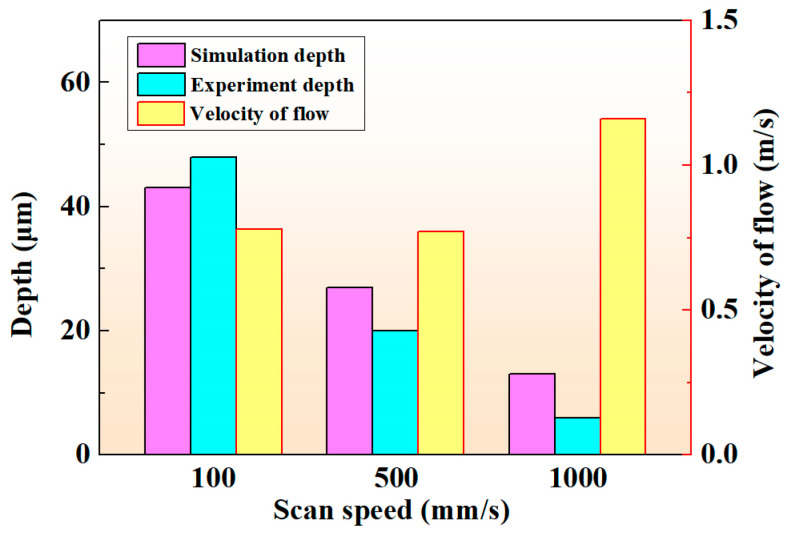
The comparison results of laser ablation depth and the variation of the flow velocity at different scanning speeds.

Figure 9 shows the experimental results of laser processing at different scanning speeds; the parameters used for the experiment were kept consistent with the parameters for the simulation. As can be seen from the figure, the height of the accumulation on both sides of the crater decreased, and the depth of the crater decreased significantly when the scanning speed increased. The comparison results of laser ablation depth between the experiment and simulation are shown in Figure 10, where the simulated results show good agreement with the experimental results. In addition, the variation of the flow velocity under different laser scanning speeds is also shown in Figure 10.

### 4.3. Effect of Average Power on the Processing Morphology

**Figure 11:**
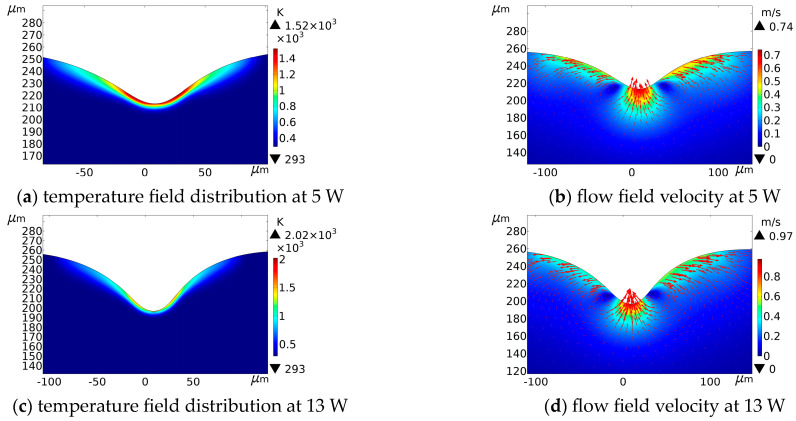
Temperature field distribution and flow field velocity at different average powers (Pulse width = 40 ns, frequency = 150 kHz, scanning speed = 100 mm/s).

The effect of laser processing on crater morphology was simulated when the average powers were 5 W, 8 W, and 13 W. Figure 3d and Figure 6 show the simulation results of temperature field distribution and flow field velocity under the average power of 8 W, respectively. The simulation results of temperature field distribution and flow field velocity under the average power of 5 W and 13 W are shown in Figure 11.

**Figure 12:**
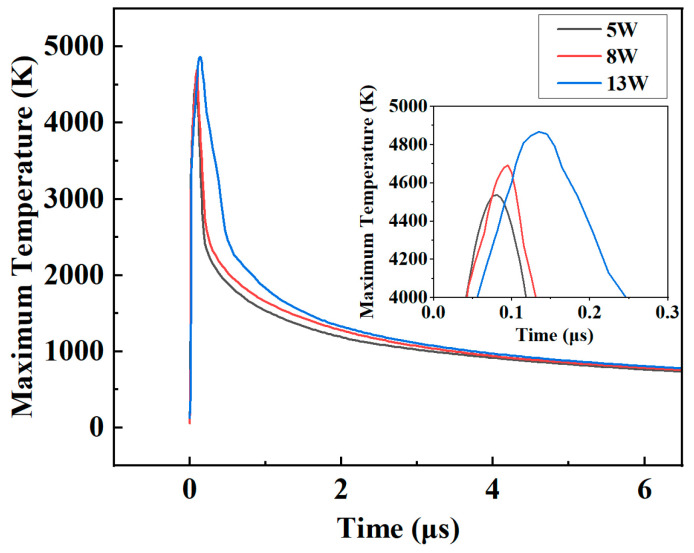
Maximum temperatures at different average power.

The results show that as the average power increased, the depth of the crater gradually increased. At the same time, the melt pool flow rate became faster, and the flow state became more intense. Moreover, the height of accumulation around the crater increased. This is because more laser energy was irradiated on the substrate surface when the average power density became higher, which increased the temperature and drove the movement of the flow field, and the evaporation of the molten material became intense. As a result, more vapor was formed, and the intensive vapor movement created a stronger recoil pressure on the molten pool, causing more molten metal to stack around the crater. Figure 12 further supports the above conclusion. In a pulse cycle, the maximum temperature becomes higher, but the drop becomes smoother when the average power increases.

**Figure 13:**
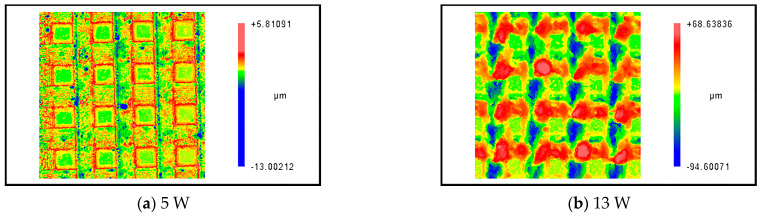
Experimental results of laser processing at different average powers (Pulse width = 40 ns, frequency = 150 kHz, scanning speed = 100 mm/s).

**Figure 14:**
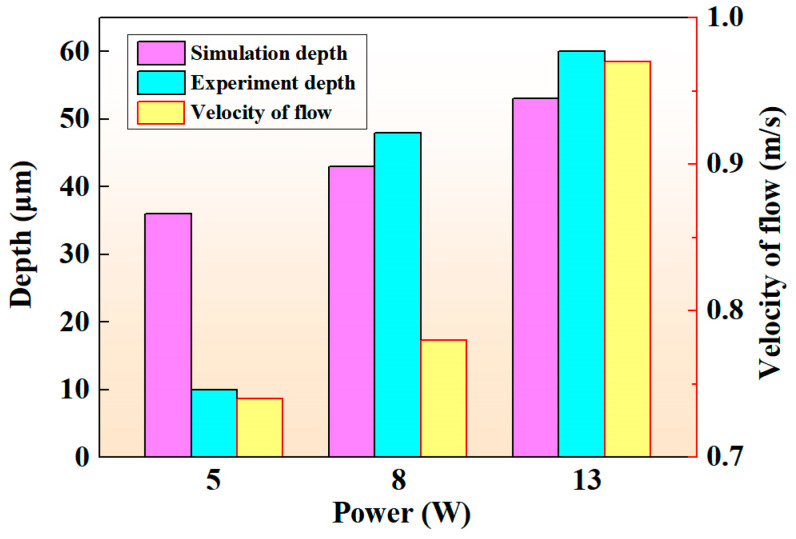
The comparison results of laser ablation depth and the variation of the flow velocity at different average power.

The experimental results are shown in Figure 13; the laser processing parameters were the same as the parameters of the simulation. The experimental results show a consistent trend with the simulation results. The ablation depth increased as the average laser power increased, and the molten material stacked more and more around the column structure. The comparison between the simulation and experimental results is shown in Figure 14. Although the variation of the experiment and simulation ablation depths shows a consistent trend, the experimental ablation depth was much smaller than that of the simulation when the average power was 5 W. This can be attributed to the surface treatment of the NdFeB material, which makes it difficult for the energy to break through the surface layer, resulting in a small ablation depth obtained in the experiment. The larger average power fabricates deeper microstructures, but the shape retention of the microstructures gets worse. As shown in Figure 13b, the ablation depth was deeper at 13 W, but the material accumulation at the edge of the columnar structure was serious. Therefore, it is necessary to balance the relationship between the ablation depth and morphology, or improve the shape retention capability by other auxiliary methods.

## 5. Conclusions

In this study, a two-dimensional simulation model of laser processing of NdFeB material was established. The temperature field distribution, microstructure morphology, and flow field velocity of the melt pool after laser processing were analyzed through simulation and experiment. The influence trends of the scanning speed and average power of laser processing on the machining morphology of microstructure were investigated. The main conclusions can be drawn as follows:

(1) A simulation model for laser processing of NdFeB materials was developed, and its accuracy was verified. Under the laser processing parameters of the average power of 8 W, pulse width of 40 ns, frequency of 150 kHz, and scanning speed of 100 mm/s, the simulation cross-section morphology showed a V-shaped profile with a crater depth of about 43 μm, which is in good agreement with the experimental results;

(2) The flow evolution of the melt pool and the formation mechanism of the microstructure in the laser processing NdFeB materials were revealed. The molten material was ejected outwards under recoil pressure, and the molten materials flowed normally to the side walls of the crater by the role of surface tension and gravity. After the temperature decreased, the molten material solidified, and a recast layer was formed at the inner wall and top of the crater, which eventually formed the processing morphology;

(3) The effect of laser scanning speed and average power on the processing morphology was investigated. When the scanning speed increased, the processing area length increased, but the ablation depth became shallow. The fluid flow intensity decreased, especially on the left side of the crater, and the melt pool flow was gentler. When the average power increased, the flow field velocity in the melt pool was more intense, and the ablation depth gradually increased.

In the future, the influence of external fields such as the electric field and the magnetic field on the processing effect in laser processing of NdFeB materials will be studied, and reveal the internal mechanism.

## Figures and Tables

**Table 1:** Physical parameters of NdFeB material.

Property	Symbol	Value	Unit
Melting temperature	*T_m_*	1811.2	K
Vaporizing temperature	*T_v_*	3135.2	K
Ambient temperature	*T_a_*	293.15	K
Solid phase density	*ρ_s_*	7500	kg/m^3^
Liquid phase density	*ρ_l_*	6500	kg/m^3^
Specific heat of solid phase	*C_ps_*	440	J/(kg∙K)
Specific heat of liquid phase	*C_pl_*	551	J/(kg∙K)
Solid phase thermal conductivity	*k_s_*	9	W/(m·K)
Liquid phase thermal conductivity	*k_l_*	7	W/(m·K)
Latent heat of fusion	*L_m_*	2.466 × 10^5^	J/kg
Latent heat of vaporization	*L_v_*	6.071 × 10^6^	J/kg
Coefficient of heat transfer	*h_1_*	15	W/(m^2^·K)
Temperature transition interval of melting	∆*T*	50	K
Dynamic viscosity of liquid phase	*μ_l_*	8 × 10^−3^	Pa·s
The surface tension of the pure metal	*γ*	1.84	N/m
Surface tension temperature coefficient	*A_γ_*	−5 × 10^4^	N/(m∙K)
Mushy zone constant	*A_m_*	10^6^	kg/m^3^∙s

**Table 2:** Laser processing parameters used for simulations.

Groups	Average Power	Scanning Speed
1	8	100, 500, 1000
2	5, 8, 13	100

## Data Availability

Data are available within the article.
